# Agroforestry benefits on dung beetle diversity of the Andean-Chocó region in Ecuador

**DOI:** 10.7717/peerj.21163

**Published:** 2026-04-23

**Authors:** Santiago Villamarin-Cortez, Betzabet Obando-Tello, Roberto Román-RR, Jorge Ari Noriega, Washington Javier Yánez Coronel, Lee Dyer

**Affiliations:** 1 Facultad de Ciencias Biológicas, Universidad Central del Ecuador, Quito, Pichincha, Ecuador; 2 Sección Invertebrados, Instituto Nacional de Biodiversidad (INABIO), Quito, Pichincha, Ecuador; 3 Department of Biology, Program in Ecology, Evolution and Conservation Biology, University of Nevada - Reno, Reno, NV, United States of America; 4 Grupo de Investigación en Ecología y Evolución en los Trópicos-EETROP, Universidad de las Americas, Quito, Pichincha, Ecuador; 5 Grupo de Agua, Salud y Ambiente/Facultad de Ingeniería, Universidad El Bosque, Bogotá, Cundinamarca, Colombia

**Keywords:** Agroecosystems, Cacao crops, Coffee crops, Monoculture, Scarabaeinae, Dung beetles, Biodiversity, Ecuador, Andean-Chocó

## Abstract

**Background:**

Conversion of natural landscapes by fragmentation and habitat modification is one of the main causes of biodiversity loss and damage to ecosystem functioning, and studies focused on modified landscapes make it possible to infer how specific land uses, such as conversion to agriculture, affect ecosystem structure and function. Here, we examine how dung beetle assemblages in the Andean-Chocó region in Ecuador vary with land use, including conventional agroecosystems with cacao and coffee production crops and reference forests at a similar elevation.

**Methods:**

We used a pitfall trapping methodology, using traps baited with human feces, each site encompassed three 60 × 60 m plots, featuring agricultural crop types (Cacao and Coffee), control sites (pristine forests), and seasonal periods (wet and dry). Sampling locations included agroforestry Cacao (AECa) and Coffee (AECo), conventional Cacao (TCa) and Coffee (TCo), and reference forests associated with Cacao (CaF) and Coffee (CoF). To characterize the total biodiversity of different treatments, we used three integrated rarefaction extrapolation curves based on three Hill numbers: species richness, the exponential of Shannon entropy, and the inverse Simpson entropy. We also used Beta diversity measured as the dissimilarity between the assemblages.

**Results:**

We collected a total of 4,380 specimens and identified 47 species of dung beetles. The areas and season with the greatest dung beetle diversity were the agroforestry cacao and reference forests and the wet season. Beta diversity was highest in Cacao ecosystems, while the Coffee plantations had the high levels of nestedness. Reference forests for the two agroecosystems exhibited higher biomass, followed by areas with agroforestry management. From these results, we can infer that agroforestry production systems in the Andean-Chocó region can contribute to the maintenance of insect species richness and ecosystem functioning and could be viable alternative conservation systems and used as biological corridors.

## Introduction

Fragmentation and habitat destruction are major causes of biodiversity loss (Reid et al., 2010). Due to the exponential growth of consumption by expanding human populations, these losses are expected to increase in the coming decades at an alarming rate (Barragán et al., 2011). Habitat transformation results in the reconfiguration of continuous natural landscapes, creating a patchwork of small, isolated, and altered habitats (Schroth et al., 2004). Understanding how biotic communities respond to such habitat modifications is essential for predicting and avoiding biodiversity loss and associated loss of ecosystem function and services (Barragán et al., 2011). Developing tools for effective management of affected natural areas (Hayes et al., 2009), such as the assessment of the functional consequences of human disturbance, will lead to better-informed conservation policies to limit biodiversity loss (Balmford & Bond, 2005).

The Andean Chocó is one of the most biologically diverse and endemic-rich regions on Earth, harboring thousands of plant and animal species, many of which are found nowhere else (Yánez-Muñoz et al., 2024). Its ecosystems, ranging from cloud forests to riverine habitats, provide critical ecological services but face intense threats from deforestation, mining, and agricultural expansion (Justicia, 2007; Waldron, Justicia & Smith, 2015).

It is often assumed that agricultural landscapes have little conservation value (Reid, Thornton & Kruska, 2005), but agroecosystems are often part of a matrix surrounding fragments of natural habitat that can vary in functional contributions to biodiversity (Evans et al., 2017). The development of agroecological or agroforestry technologies and systems has increasingly focused on the conservation or regeneration of biodiversity, soil, water, and other important resources (Altieri, 1999). In the Neotropics, cacao and coffee agroforestry systems that include forest fragments are among many land use options that could provide some support for maintaining tropical forest biodiversity (Noriega et al., 2012), as crops in the middle of forests cause less impact (Rice & Greenberg, 2000). Land-use dynamics in the Ecuadorian Chocó are driven by the conversion of montane forest into pastures, agricultural fields, and agro-pastoral mosaics (Noh et al., 2022).

These transitions often involve rotations among pasture, annual crops, and fallow cycles, with a net expansion of pasture at the expense of both forest and agriculture, contributing to deforestation and fragmentation. In the Mashpi drainage, the landscape now comprises intact cloud forest, organic agroforestry farms, and extensive palmito (*Bactris gasipaes*) monocultures (Morabowen, Crespo-Pérez & Ríos-Touma, 2019), the latter involving complete forest removal and agrochemical use that alter streams and reduce aquatic biodiversity. By contrast, traditional cocoa agroforestry retains shade-tree canopies that provide habitat for wildlife (Rice & Greenberg, 2000; Ruf & Schroth, 2004; Bhagwat et al., 2008), maintain carbon storage (Gockowski & Sonwa, 2010), and supply ecosystem services locally and globally (Schroth & Harvey, 2007; Gockowski & Sonwa, 2010).

Agroforestry approaches hold promise for bolstering biodiversity conservation within agricultural and forested areas by augmenting structural diversity and enriching habitat and landscape heterogeneity (Lopez-Bedoya et al., 2022; Santos et al., 2022). To contribute to maintaining diversity, these ecosystems must retain a sufficient amount of tree cover (Celi-Santos & Philpott, 2019; Holzschuh, Steffan-Dewenter & Tscharntke, 2008; Kremen et al., 2004), add substantial organic material to the soil, help maintain a certain degree of connectivity among landscapes, and include conservation planning (Harvey et al., 2008). In contrast, conventional crop techniques may support lower levels of biological diversity and relatively few forest-dependent organisms, as they convert a forest into a crop (Cozim-Melges et al., 2024).

Insects are declining in multiple biomes (Cardoso et al., 2020; Wagner, 2020; Wagner et al., 2021). As key mediators of many ecosystem processes, extinctions could produce cascading effects on entire biotic communities (Coleman & Hendrix, 2000; Dyer & Letourneau, 2013). Insects in particular are frequently used as bioindicators, as insect declines cause or indicate various types of ecosystem degradation (Audino, Louzada & Comita, 2014; Noriega et al., 2021a) and are often severely affected by landscape changes over rapid timescales (Dunn, 2004; Samways, 2005; Noriega et al., 2021b). Despite this, the response of insects to agroforestry can be relatively limited (Shahabuddin, Schulze & Tscharntke, 2005; Barragán et al., 2011; Neita & Escobar, 2012; De Farias & Hernández, 2017; Santos-Heredia et al., 2018), but it is important to better understand how the composition and abundance of biodiversity differ across agroecosystems, which fluctuate in age, structure, and management (Altieri, 1999).

Dung beetles (mostly Scarabaeinae and Aphodiinae) are an ideal taxonomic and functional group for the study of the relationships between anthropogenic disturbances, assemblage structure, and insect declines (Favila & Halffter, 1997; Nichols et al., 2008; Otavo, Parrado-Rosselli & Noriega, 2013; Villamarín-Cortez, 2013). These beetles have a wide global distribution and are a diverse and abundant group in both tropical and warm temperate ecosystems (Hanski & Cambefort, 1991; Villamarin-Cortez et al., 2022; Moctezuma, Halffter & Escobar, 2024). Dung beetles also have well-known ecological roles and a relatively stable taxonomy, due to their distinctive and recognizable features, making them well-studied compared to some other insect groups (Philips, Pretorius & Scholtz, 2004) and ideal bioindicators for conservation (Carvajal, Villamarín-Cortez & Ortega, 2011; Villamarín-Cortez, 2010; Larsen, 2004; Celi et al., 2004; Hamel-Leigue, Herzog & Mann, 2008).

Therefore, they play a pivotal role in maintaining ecosystem functioning through nutrient recycling, soil aeration, and parasite suppression, thereby influencing ecosystem productivity and animal health. However, their activity is closely tied to rainfall patterns, with emergence and dormancy phases regulated by short- and long-term precipitation dynamics (Simba et al., 2022; Davis, Deschodt & Scholtz, 2020). Consequently, seasonal fluctuations, particularly the pronounced decline in abundance and species richness from the rainy to the dry season, can substantially diminish dung beetle-mediated ecosystem services such as dung burial and secondary seed dispersal (Andresen, 2005; Tshikae, Davis & Scholtz, 2013; Hernández et al., 2014; Correa et al., 2024). These seasonal reductions are further shaped by interactions with land-use practices, including vegetation cover changes (Correa et al., 2021) and livestock management (Harvey et al., 2008; Correa et al., 2024), which alter community composition and functional diversity. The loss of forest-associated and functionally specialized species during dry periods may shift assemblages toward generalist dominance, thereby weakening key ecological processes.

Dung beetles associated with agricultural environments promote greater appreciation of the ecosystem functions provided by these organisms because their ecosystem functions are better understood, including their relationship with soil dynamics (Farias & Hernández, 2017). Both adults and larvae feed mainly on feces of omnivorous mammals and carrion, and this foraging behavior contributes to the recycling of nutrients through the burial of feces and carrion in the soil, pest population reduction, bioturbation (oxygenation of the soil), pollination, secondary seed dispersal, and reducing greenhouse gas emissions (Nichols et al., 2008; Slade et al., 2016).

Our study on the structure and composition of dung beetle assemblages was guided by the question of how agricultural management practices affect insect diversity; and was focused on dung beetles in a landscape with a combination of different systems: forests, cacao, and coffee plots in the Andean-Chocó region in the Nor-occidental region of Ecuador. We tested these two specific hypotheses: (1) Cacao and Coffee agroforestry crops exhibit levels of dung beetle diversity comparable to adjacent reference forests; (2) Tropical Forest beta diversity associated to seasonality is maintained in agroecosystems, with these systems supporting different beetle assemblages in wet *versus* dry seasons. We predicted that shade-grown cacao and coffee plots provide sufficient structural complexity and resources to support diverse beetle communities, and that rainfall-driven changes in resource availability would generate seasonal shifts in species composition.

## Materials & Methods

### Study area and field sampling

The study was carried out in the northwestern Andean slope in Ecuador, characterized by two ecosystems: evergreen montane forest and evergreen foothill forest, both located on Western Cordillera of the Andes. The study sites are influenced by the Chocó bioregion, identified as a hotspot of biodiversity (Myers et al., 2000), and characterized by a subtropical humid climate with 800 mm mean annual precipitation, with a mild dry season that lasts for 6 months (Sierra, 1999). All specimens were collected with approval from the Ministry of the Environment of Ecuador under the permit No. MAE-DNB-CM-2016-0045 and the scientific support of the National Institute of Biodiversity.

**Figure 1:**
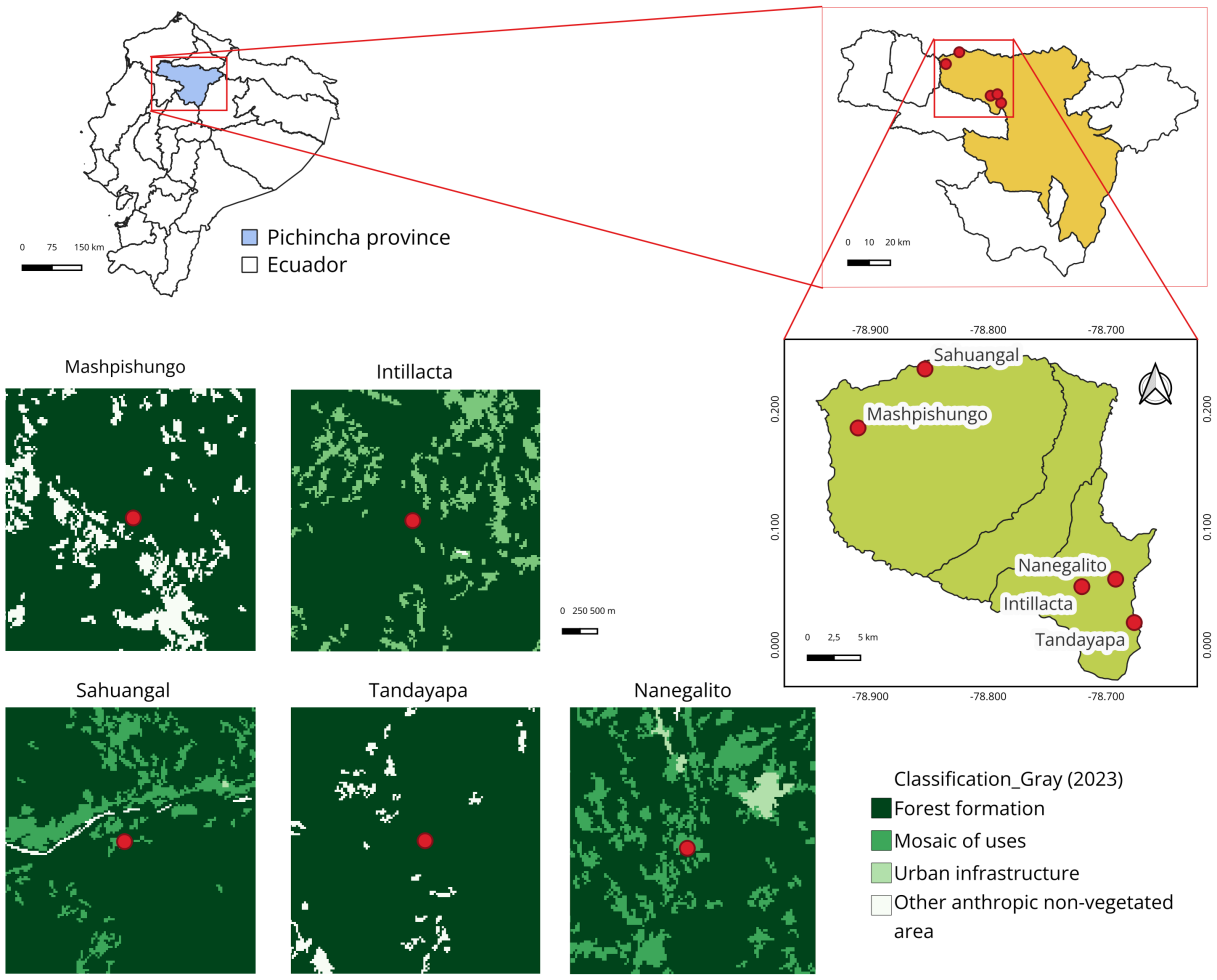
Study sites. Study area showing the location of the sampling windows at the Chocó Region in Northern Ecuador.

We selected six sampling sites representing two agricultural crop systems (Cacao = Ca and coffee = Co), each with agroforestry (AE) and conventional (T) management, along with their associated pristine forest controls (CaF and CoF). The sites were: AECa in Mashpishungo (0.1840°N, −78.9104°W), TCa in Sahuangal (0.2340°N, −78.8536°W) and Nanegalito (0.0558°N, −78.6919°W), AECo in Intillacta (0.0494°N, −78.7205°W), and TCo in Nanegalito (0.0558°N, −78.6919°W); see Fig. 1. Sampling was performed during both wet (November–January) and dry (June–August) seasons across an elevational gradient of 600–1,600 m. The authors thank Miguel Verkade, Margarita Chiriboga, Hernán Zúñiga, and Kléver Gutiérrez for allowing the study on their lands.

These factors were analyzed using a crossed design with site (three levels: agroecological, conventional, and forest control) and season (two levels: wet and dry) as fixed treatment variables. Within each site, we established three 60 × 60 m quadrats separated by at least 500 m to minimize spatial dependence among sampling units. Inside each quadrat, we deployed a cluster of nine pitfall traps baited with human feces, arranged in a 30-m grid. This inter-trap spacing follows established protocols in dung beetle ecology (*e.g.*, Larsen & Forsyth, 2005) and is widely considered sufficient to ensure assemblage independence due to the limited attraction radius of baited traps and the ephemeral nature of dung resources. Over two decades of standardized regional sampling using this configuration have shown that it reliably captures local assemblage structure while minimizing the risk of short-range spatial autocorrelation. For this reason, and because our analyses focused on treatment-level effects rather than fine-scale spatial patterns, we did not conduct additional statistical tests of spatial autocorrelation among traps or quadrats. The same design was implemented in both cacao and coffee systems, resulting in a total of 324 traps (162 per crop across both seasons).

Pitfall traps were baited with 25 g of fresh human feces, gauze wrapped and suspended above 12-ounce plastic containers buried at ground level (Newton & Peck, 1975; Morón & Terrón, 1984; Lobo, Lumaret & Jay-Robert, 1998; Halffter & Favila, 1993; Mora-Aguilar et al., 2023). These traps were monitored once a day and removed after 48 h. Beetles were collected and placed in 32-ounce whirl-pack collection bags filled with 96% ethanol. Once in the laboratory, the beetle samples were cleaned and identified up to species using specialized taxonomic keys (Jessop, 1984; Génier, 1998; Génier, 2009; Sarmiento-Garcés & Amat-García, 2009; Edmonds & Zídek, 2010); and compared with curated identified material at the entomological scientific collection from the National Institute of Biodiversity of Ecuador (INABIO).

Beetle biomass was quantified for the most representative species for the entire assemblage within each habitat, following the procedures outlined by Davis, Huijbregts & Krikken (2000) and Lobo (1993). Five individuals per species were randomly selected, oven-dried at 150 °C for 12 h and subsequently weighed using a precision (0.0001 g) electronic balance (Sartorius BP 210S), to determine the mean individual biomass for each species. Species represented by fewer individuals were excluded from the analysis.

### Data analysis

To characterize the total biodiversity of different treatments, we used three integrated rarefaction extrapolation curves based on three Hill numbers: species richness, the exponential of Shannon entropy, and the inverse Simpson entropy, corresponding to Hill numbers of order *q* = 0 (0D), *q* = 1 (1D) and *q* = 2 (2D), respectively (Hill, 1973; Chao et al., 2014). The diversity analysis was used to quantify local diversity (*α*) of dung beetle assemblages for each treatment level combination and to determine if there are diversity differences in the dung beetle assemblages across coffee and cacao agroecosystems and reference forests. These diversity measures include the most commonly used Hill numbers (although any number could be used) and include a good range of differential weighting of rare species; they also have the useful attribute, and its units are always in effective number of species (Jost, 2006). To quantify diversity profiles, we used iNext Online, based on Chao et al. (2016).

Prior to any inferential analysis, we evaluated the suitability of the alpha diversity metrics for parametric modeling by examining their distributional properties without fitting statistical models. For continuous diversity measures—namely the exponential of Shannon entropy (Hill number 1D) and the inverse of Simpson concentration (Hill number 2D)—we assessed approximate normality using histograms and quantile–quantile (Q–Q) plots and evaluated homoscedasticity by visually comparing variance among treatment groups. Therefore, to assess differences in dung beetle biodiversity among crop types, we conducted a one-way ANOVA and reported effect sizes using eta squared (*η*^2^). Eta squared was chosen because it provides a standardized measure of the proportion of variance in biodiversity explained by crop type, offering insight into the magnitude of observed differences beyond statistical significance. All analyses were performed using R and its package stats (R Core Team, 2024).

To assess differences in beetle assemblages between seasons, we calculated species diversity using Shannon’s entropy index (H’). Diversity values were computed for each site and season, and comparisons were made between the wet and dry periods to evaluate seasonal variation in community structure.

Beta diversity, in dung beetle species composition across treatment level combinations was measured as the dissimilarity between the assemblages. This function computes the overall dissimilarity (measured as Jaccard dissimilarity) between treatment levels and partitions it into its turnover and nestedness-resultant components (Baselga, 2010; Baselga, 2012). The multiple-site Jaccard dissimilarity was partitioned using the package Betapart v1.3 (Baselga & Orme, 2012) to determine whether the ecological differences between sampling units resulted from species turnover (BRepl) or nestedness (BRich). Turnover measures the replacement of species between sites caused by environmental differences, disturbance, or competition. Nestedness is a loss of species between sites, usually due to differences in local conditions or ecological niches, where the species-poorer site contains a subset of the species present in the species-richer site (Legendre, 2014). We performed a non-metric multidimensional scaling (NMDS) analysis using total beta diversity values to visualize community dissimilarities among crop types (Chase et al., 2011). These two components reflect the substitution of some species by others across sites (turnover), and the loss or gain of species in a nested pattern (nestedness-resultant component), respectively (Baselga, Bonthoux & Balent, 2015). These measures were estimated using R 3.5.0 (R Core Team, 2024), and the betapart (Baselga & Orme, 2012) package.

## Results

### Dung beetle assemblage structure and diversity

A total of 4,380 dung beetles, representing 47 species, 17 genera, and seven tribes were collected from all sites combined (Table 1). Specifically, 2,598 individuals from 38 species, 16 genera, and seven tribes were identified in cacao agroforestry systems, conventional cacao crops, and adjacent reference forests. Meanwhile in coffee plantations and associated reference forests, we collected a total of 1,782 dung beetle specimens, representing 18 species, 10 genera, and seven tribes.

**Table 1:** Dung beetle species list. List of species by site (Cacao Forest: CaF, Agroforestry Cacao: AECa, Conventional Cacao: TCa, Coffee Forest: CoF, Agroforestry Coffee: AECo, and Conventional Coffee: TCo) and seasonality (Dry season: D and Wet season: W).

		**CaF**		**AECa**		**TCa**		**CoF**		**AECo**		**TCo**	
**Tribe**	**Species**	**D**	**W**	**D**	**W**	**D**	**W**	**D**	**W**	**D**	**W**	**D**	**W**
Ateuchini	*Ateuchus* sp.								11				
	*Bdelyrus* sp.					1							
	*Scatimus* sp.								10				
Coprini	*Canthidium aurifex. Bates 1887*				1								
	*Canthidium centrale Boucomont, 1928*	2	1				1						
	*Canthidium bicolor Boucomont, 1928*	1											
	*Canthidium haroldi. Preudhomme de Borre 1886*	2											
	*Canthidium pseudaurifex. Balthasar 1939*	2	5	1									
	*Canthidium splendidum.Preudhomme de Borre 1886*	7	2	4	4								
	*Canthidium* sp. 9	1											
	*Canthidium* sp. 10	2	2										
	*Ontherus compressicornis. Luederwaldt, 1931*	9						4		1		3	
	*Ontherus diabolicus. Génier, 1996*								164		140		135
	*Ontherus trituberculatus. Balthasar 1938*		19		1								
	*Uroxys brachialis Arrow 1933*							19	153				
	*Uroxys* sp.	1	1							2			
Deltochilini	*Anomiopus* sp.	39	45										
	*Canthon delicatulus. Balthasar 1939*				2	336	95						
	*Deltochilum aff. batesi*								4	12	34		
	*Deltochilum aff. cristinae*							1				1	
	*Deltochilum gibbosum*	11	17										
	*Deltochilum loperae. González & Molano, 2009*	1	3		1								
	*Deltochilum aff. luderwalti*							1	4		1		
	*Deltochilum aff. parile*	14		11	6	24	9						
	*Scybalocanthon trimaculatus. Schmidt, 1922*	4	1		1								
Dichotomini	*Dichotomius divergens Luederwaldt 1923*	27	98	9	42		1		124	1	75	2	37
	*Dichotomius fortepunctatus Luederwaldt, 1923*	693	642	6	46		8						
Oniticellini	*Eurysternus caribaeus. Herbst, 1789*	1	1										
	*Eurysternus marmoreus.Castelnau, 1840*							1			1		
	*Eurysternus plebejus. Harold, 1880*					1	2						
Onthophagini	*Onthophagus acuminatus. Harold, 1880*		128		11		6						
	*Onthophagus belorhinus. Bates, 1887*	1											
	*Onthophagus lojanus. Balthasar, 1939*		17		60		2						
	*Onthophagus aff. nabeleki*	3		9		5	4	15		84	18	160	3
	*Onthophagus group Dicranius*								66		136		272
	*Onthophagus aff. rhinolophus*			3				2					
	*Onthophagus aff. stockwelli*	2	1										
	*Onthophagus* sp. 7				3	3				3		2	
	*Onthophagus* sp. 8					2							
	*Onthophagus* sp. 9			5									
	*Onthophagus* sp. 10			2			3	1	11	11	30	5	18
Phanaeini	*Coprophanaeus conocephalus. d’Olsoufieff, 1924*				2		1						
	*Oxysternon conspicillatum.Weber, 1801*	1	20										3
	*Oxysternon silenus. d’Olsoufieff, 1924*	1			3		1						
	*Phanaeus funereus Balthasar, 1939*	9	13		9					1			
	*Sulcophanaeus miyashitai. Arnaud, 2002*	1											
	Abundance	835	1,016	50	192	372	133	44	547	115	435	173	468
	Richness S’	24	18	9	15	7	12	8	9	8	8	6	6

**Figure 2:**
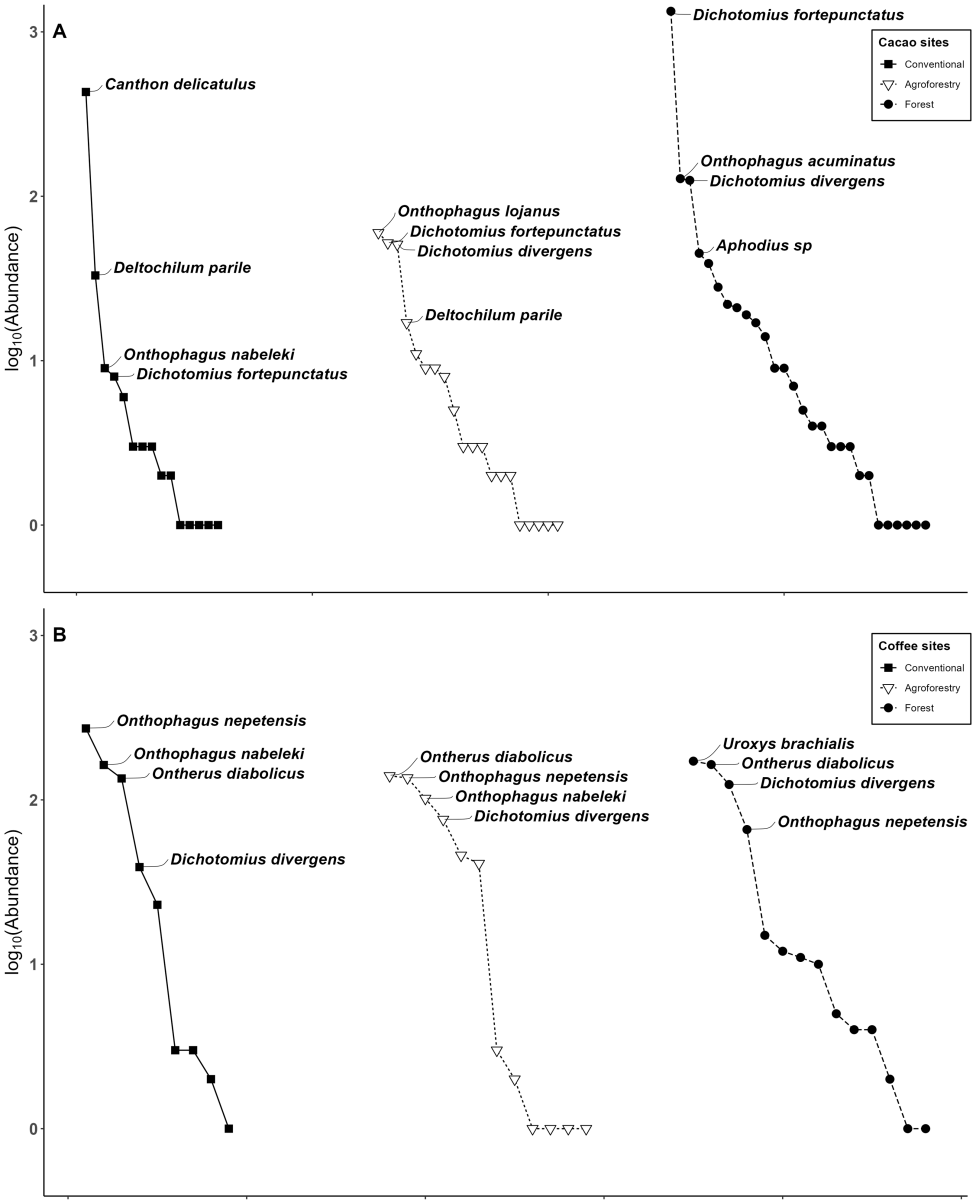
Dominance. Dung beetle species’ dominance assemblages from (A) Cacao sites and (B) Coffee sites, showing the most important species.

Species assemblages showed significant variation across all habitat types (Fig. 2). During the dry season, the dung beetle assemblage in cacao agroforestry systems was dominated by *Deltochilum parile* Bates, 1887 (Deltochilini, *n* = 11), whereas *Onthophagus lojanus* Balthasar, 1939 (Onthophagini, *n* = 60) dominated during the wet season (Table 1). In conventional cacao crops, *Canthon delicatulus* Balthasar, 1939 was the most abundant species in both seasons (Deltochilini, Dry: *n* = 336; Wet: *n* = 95, Table 1). In the control forest, *Dichotomius fortepunctatus* (Luederwaldt, 1923) (Dichotomiini) was the dominant species, with 693 individuals recorded during the dry season and 642 during the wet season (Table 1, Fig. 2A).

Coffee control forest assemblages showed *Uroxys brachialis* Arrow, 1933 (*n* = 175) as the overall dominant species (Fig. 2B). In agroforestry coffee systems, *O. diabolicus* Harold, 1877, was the most dominant species (*n* = 140). In conventional coffee plantations, *Onthophagus sp* (*group Dicranius*) was the dominant species (*n* = 272), see Table 1, Fig. 2B.

**Figure 3:**
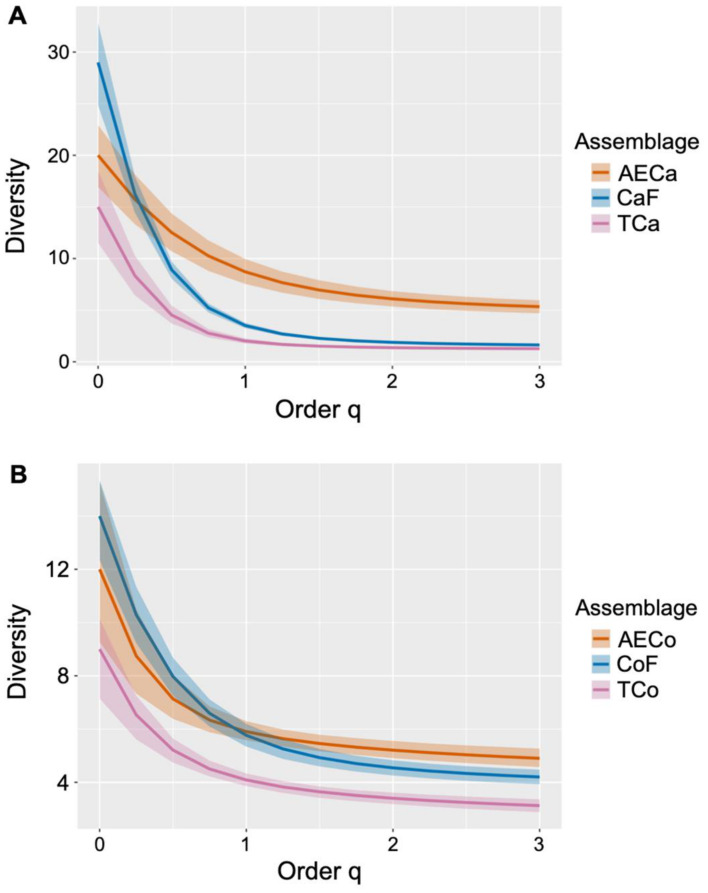
Diversity analysis. Diversity profiles based on Hill’s numbers of order *q* = 0 Richness, *q* = 1 Exponential of the Shannon Entropy, and *q* = 2 Inverse Simpson Concentration, for (A) cacao treatments: Agroforestry Cacao (AECa), Cacao Forest (CaF), and Conventional Cacao (Tca) and (B) coffee treatments: Agroforestry Coffee (AECo), Coffee Forest (CoF), and Conventional Coffee (Tco).

Species richness (S’) was higher in forests around areas of conventional cacao and agroforestry crops (*η*^2^ = 0.78; *F* = 15.39; *df* = 8; *p* = 6.25E−13; Fig. 3A). For coffee production, conventional coffee assemblages had lower richness than the reference forests and agroforestry production (*η*^2^ = 0.38; *F* = 3.96, *df* = 8, *p* = 0.0006; Fig. 3B). According to the effective number of species, agroforestry cacao assemblages had the highest Shannon Entropy (q_1_ = 8) and the highest Inverse Simpson Concentration (q_2_ = 6; Fig. 3A). Coffee agroforestry crops had the greatest diversity, with higher Exponential of Shannon Entropy (q_1_ = 6) and Inverse Simpson Concentration (q_2_ = 5) than the reference forest (q_1_ = 5.8, q2 = 4.3) and conventional cacao (q_1_ = 4.1, q_2_ = 3; Fig. 3B).

**Figure 4:**
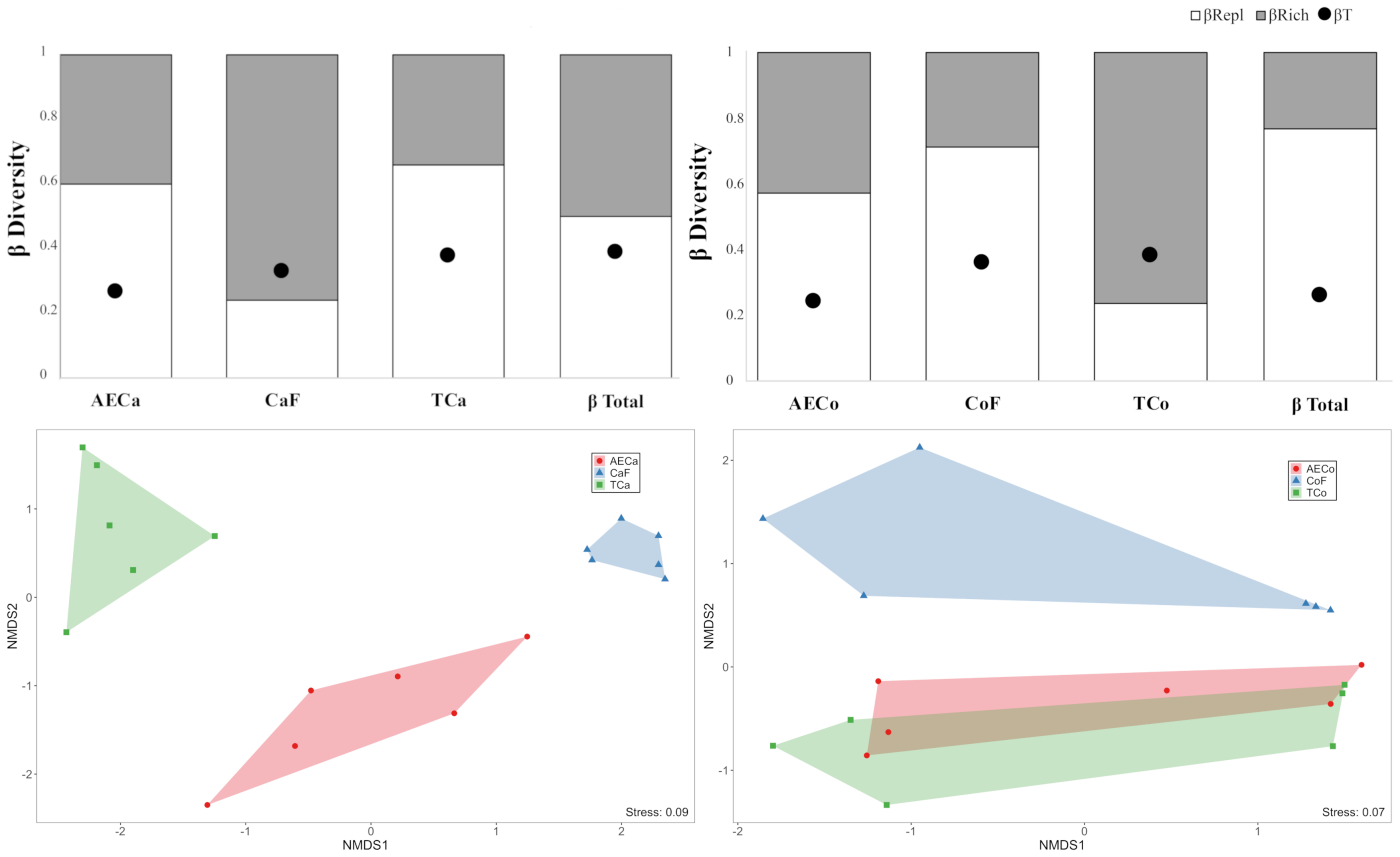
Beta diversity analysis. Beta diversity within crop treatments (A) Cacao and (B) Coffee; black dots: Jaccard beta diversity (b T) between all treatments, white bars: Percentage contribution to species replacement or turnover (b Repl) to beta diversity, and grey bars: Percentage contribution to species-nestedness (b Rich) to beta diversity. (C & D) Non-metric multidimentional scaling ordination for (C) Cacao and (D) Coffee treatments; AECa/o, Agroforestry; Ca/oF, Forest Control; TCa/o conventional.

Beta diversity, calculated using the multiple-site Jaccard index, showed moderate dissimilarity in cacao sites (*β* Total = 0.40, Fig. 4A), with equal contributions from species turnover and nestedness. Coffee sites had lower beta diversity (*β* Total = 0.30, Fig. 4B), driven primarily by species replacement. These results indicate that species replacement (BRepl) is the dominant component that explains the differences in beetle composition between management systems and their control forests. However, while turnover is the main process, the presence of a nesting component (BRich) is not negligible, especially in traditional systems.

The NMDS analysis, with a stress level of 0.09 for Cacao and 0.07 shows a distinctive clustering of communities. In the space defined by the NMDS1 and NMDS2 axes, it is evident that the control forests (CaF and CoF) form separate and compact groups, indicating that Dung Beetle communities are consistent and distinct from those of the agricultural systems.

The agroecological systems (AECa and AECo) and the traditional systems (TCa and TCo) are positioned in intermediate locations within the NMDS gradient. However, greater similarity in composition is observed between the agroecological systems and their respective control forests, compared to the traditional systems (Figs. 4C and 4D).

### Dung beetle seasonality and biomass

**Figure 5:**
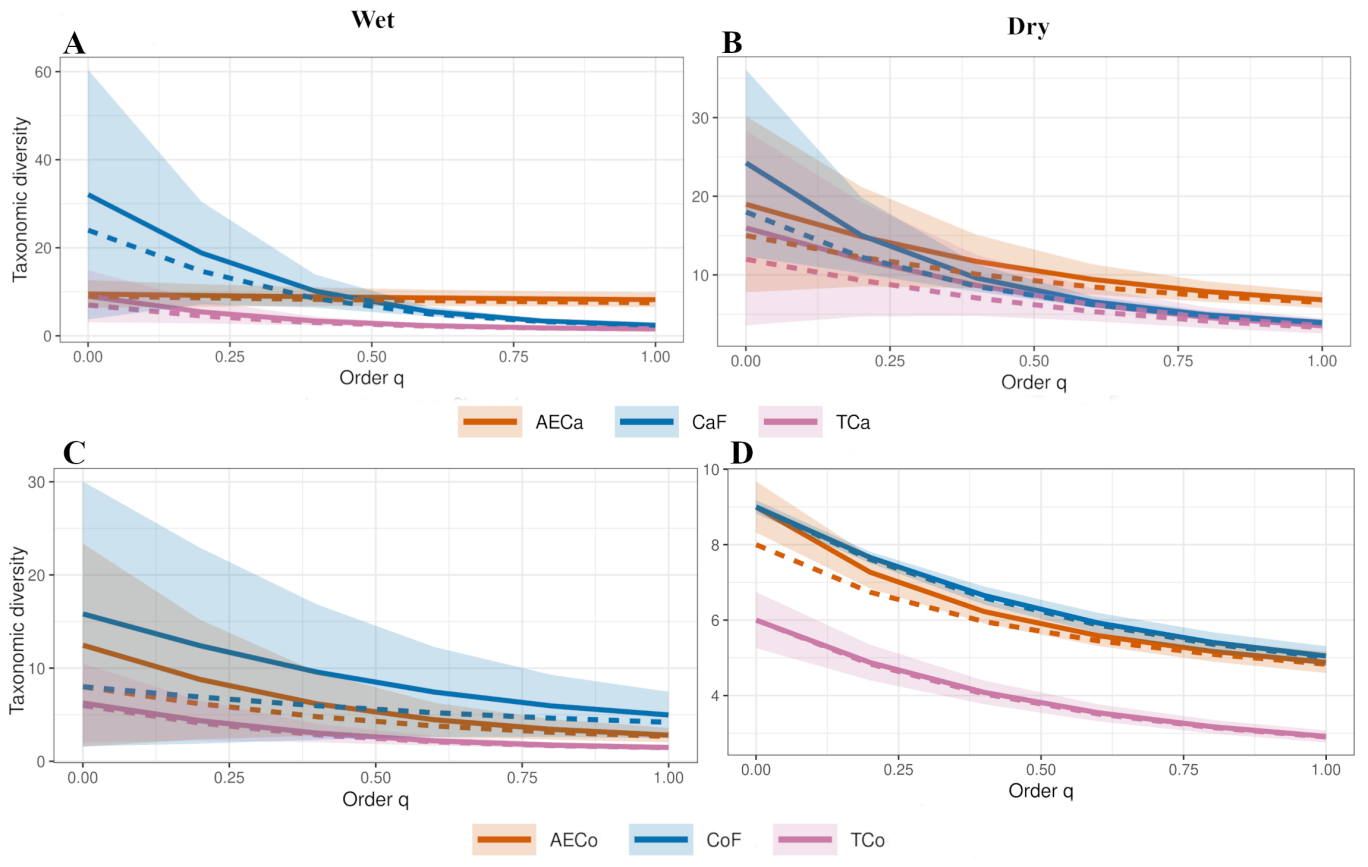
Seasonality analysis. Diversity profiles based on Hill’s numbers of order *q* = 0 Richness, *q* = 1 Exponential of the Shannon Entropy, according to seasonality for (A) wet season for Cacao treatments: Agroforestry Cacao (AECa), Cacao Forest (CaF), and Conventional Cacao (Tca) and (B) dry season for Cacao treatments. (C) Wet season for Coffee treatments: Agroforestry Coffee (AECo), Coffee Forest (CoF), and Conventional Coffee (Tco) and (D) dry season for Coffee Systems.

The diversity profiles across seasons and land-use systems reveal distinct patterns in the taxonomic diversity of dung beetle assemblages. During the wet season (Figs. 5A and 5C), species richness (*q* = 0) was highest in forest controls (CaF: 28 species; CoF: 25 species), followed by agroecological systems (AECa: 22; AECo: 20), and was lowest in traditional plantations (TCa: 15; TCo: 14). This hierarchy was largely maintained in the exponential of Shannon entropy (*q* = 1), with wet season values for forests (CaF: 18.2; CoF: 16.8) exceeding those of agroecological (AECa: 14.1; AECo: 12.9) and traditional systems (TCa: 10.3; TCo: 9.7). In the dry season (Figs. 5B and 5D), all systems exhibited a marked decline in diversity metrics, but the rank order of treatments persisted. Dry-season richness was substantially reduced in traditional coffee (TCo: eight species) compared to its adjacent forest (CoF: 18 species), while agroecological coffee (AECo: 13 species) showed intermediate resilience. Consequently, the disparity in diversity profiles between forest controls and agricultural systems was most pronounced during the dry season, particularly at *q* = 0.

**Figure 6:**
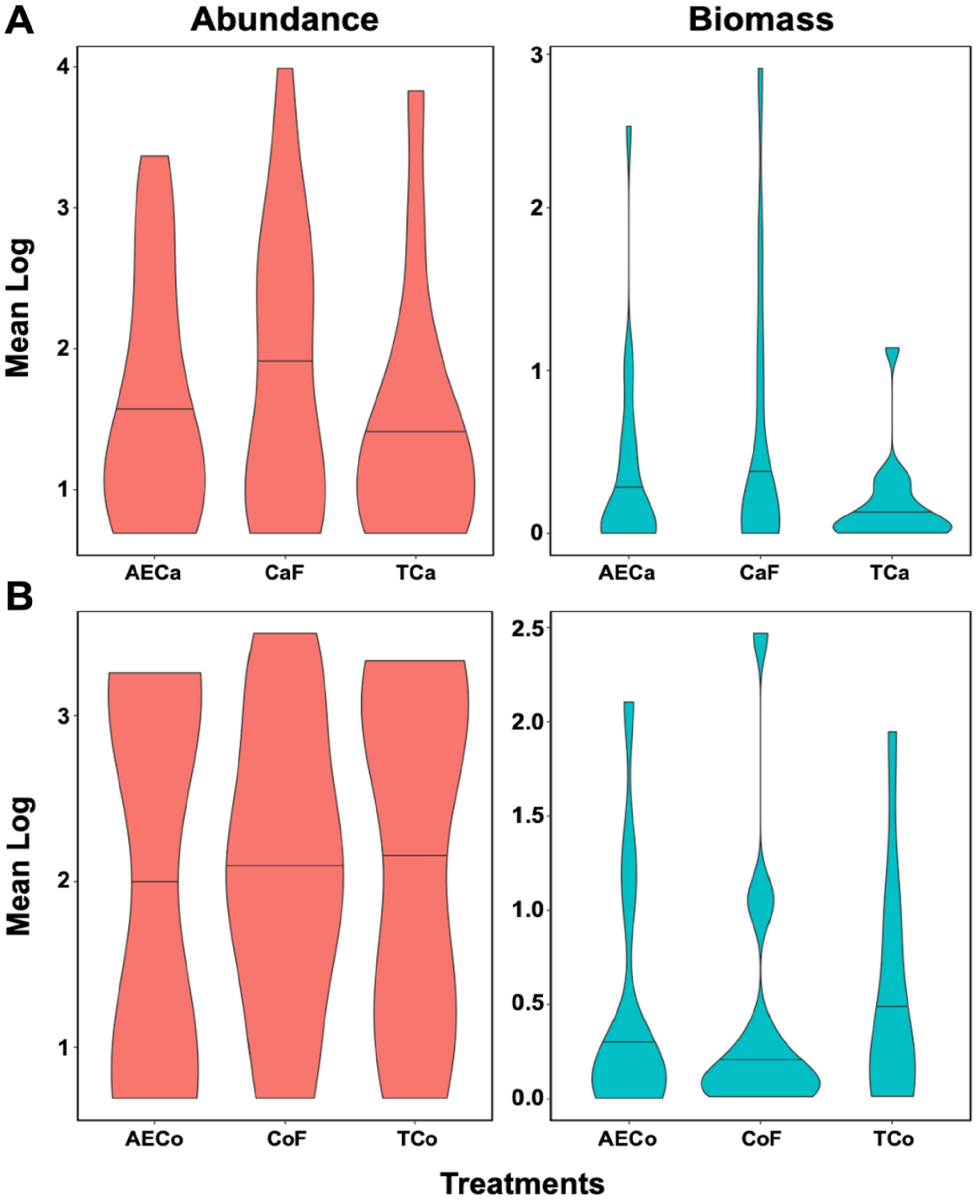
Abundance/biomass analysis. Abundance and biomass mean for (A) Cacao treatments (Cacao Forest: CaF, Agroforestry Cacao: AECa, and Conventional Cacao: TCa) and (B) Coffee treatments (Coffee Forest: CoF, Agroforestry Coffee: AECo, and Conventional Coffee: TCo).

Dung beetle abundance was greatest in forests near cacao crops, but conventional cacao sites had the highest variability, with a significant difference between sample means (*p* < 0.001, Fig. 6A). In coffee crops, conventional systems had more specimens, though differences in means were not significant (Table 2, Fig. 6B).

**Table 2:** Abundance and biomass. Dung beetle abundance and biomass across cacao and coffee management systems.

**Sites**	**Abundance mean (ind ± SD)**	**Biomass mean (g ± SD)**
CaF	64 ± 247	1.326 ± 3.27
AECa	12 ± 19	0.940 ± 2.55
TCa	34 ± 110	0.277 ± 0.55
CoF	42 ± 63	0.449 ± 0.66
AECo	46 ± 55	1.089 ± 2.11
TCo	97 ± 71	1.113 ± 1.93

**Notes.**

CaF forest controls near cacao crops AECa agroecological cacao TCa traditional cacao CoF forest controls near coffee crops AECo agroecological coffee TCo traditional coffee

These results indicate a clear hierarchical pattern. The forest control sites (CaF and CoF) consistently exhibit the highest and most broadly distributed abundances, as evidenced by their tall and wide violins, suggesting these habitats support robust and dense populations. In contrast, the conventional cropping systems (TCa and TCo) show the most constrained abundances, with notably narrower violins, indicating lower and less variable population densities. The agroecological systems (AECa and AECo) demonstrate an intermediate profile, with violins that are generally wider and taller than their conventional counterparts but not reaching the dimensions of the forests.

Biomass was highest in control forests near cacao plantations (50.73 g), with agroforestry and conventional crops showing lower values (17.83 g and 3.87 g, respectively, *p* < 0.001, Fig. 6A). Biomass in coffee treatments showed no significant variation (*p* = 0.525). The forest controls display the highest median biomass values and the widest distributions, underscoring their role in supporting larger-bodied individuals and a more functionally diverse assemblage (Fig. 6B). The conventional systems (TCa, TCo) consistently show the lowest median biomass and the most restricted distributions. Notably, the agroecological systems, particularly AECo, appear to support a biomass distribution that more closely approximates the forest condition than what is observed for abundance (Fig. 6B).

## Discussion

Understanding how biotic communities respond to habitat transformation is essential for developing tools for effective management to avoid biodiversity loss. It is frequently assumed that agricultural landscapes have little conservation value, but in the Neotropics, Cacao and Coffee agroecosystems are often part of a matrix surrounded by forest fragments that could provide support in maintaining tropical biodiversity. Our main result reveals that species richness, abundance, and biomass were significantly higher in forests near agroforestry Cacao and Coffee crops and that seasonality is present in all systems.

### Dung beetle’s assemblage composition and structure

There were notable differences in dung beetle composition across different Cacao and Coffee-associated ecosystems. Lower beetle densities associated with conventional Cacao and Coffee crops were likely a result of low densities of the focal crops, which affects important abiotic parameters, such as soil hardness and temperature (Nichols et al., 2008). Dung beetle abundance in agroforestry systems was positively correlated with biomass, in contrast to the conventional cultivation systems that had greater abundances of lower biomass species (Montoya-Molina et al., 2015; Klein, Steffan-Dewenter & Tscharntke, 2002; Somarriba et al., 2004; Villada-Bedoya et al., 2016), which reduces successful breeding and manure burial (Maldonado et al., 2018). Indeed, conventional Cacao and Coffee crops contribute to lower levels of biological diversity, in particular, due to the lack of food provided by mammals, which is driven by the absence of trees and shaded areas (Rice & Greenberg, 2000; Harvey, Gonzalez & Somarriba, 2006), the same phenomenon has been characterized in pastures (Villamarín-Cortez, 2010). In Cacao and Coffee agroforestry systems, the arrangement of mixed crops with other fruit trees and dense plant cover enhances soil conditions and understory temperature, which could provide good conditions for dung beetles (Harvey, Gonzalez & Somarriba, 2006; Barlow et al., 2007; Nichols et al., 2008; Nichols et al., 2009; Chung et al., 2009). The overall increase in areas with intensive anthropogenic disturbances acted as an environmental filter (Spector & Ayzama, 2003; Gardner et al., 2008), allowing small species, such as *C. delicatulus*, to act as a stress-tolerant dung beetle able to survive, have wide distributions, high abundances, and dominance in conventional crops. According to Carvajal, Villamarín-Cortez & Ortega (2011), these beetles are highly adaptable, becoming dominant in open areas, allowing them to use of small patches of plant shade. Forests adjacent to agricultural systems may provide tree cover that influences species dispersal capacity and diversity in these productive ecosystems (Lumaret & Kirk, 1987; Halffter & Matthews, 1966; Lobo, Lumaret & Jay-Robert, 1998).

Despite altering the forest matrix, agroforestry systems can serve as valuable refuges for biodiversity (Harvey, Gonzalez & Somarriba, 2006; Barlow et al., 2007; Nichols et al., 2008; Nichols et al., 2009; Chung et al., 2009), as demonstrated in this study. For instance, dung beetle species typically associated with forest interiors, such as those of the genus *Deltochilum*, were observed utilizing agroforestry systems as feeding grounds. The dung beetle assemblage in these agroforestry systems closely resembled that of forest controls, albeit with reduced abundance. All these results suggest that agroforestry systems can function as effective ecological corridors between forest fragments, a role not observed in conventional crop systems, which lacked forest-associated species. Furthermore, conventional crops exhibited significantly lower alpha diversity compared to agroforestry systems. This pattern of diversity aligns with the intermediate disturbance hypothesis (Fox, 1979), which posits that moderate levels of disturbance can sustain higher biodiversity by preventing competitive exclusion while maintaining habitat heterogeneity.

### Dung beetle’s assemblage diversity, abundance, and biomass

Species turnover was the primary driver of beta diversity and complementarity of dung beetle assemblages found in cacao treatments, whereas nestedness drove beta diversity in coffee treatments. There were up to 15 species not shared between treatments in Cacao and up to three species not shared in coffee treatments. The pattern of beta diversity found in the results shows moderate dissimilarity among the cacao sites, with equal contributions between species turnover and nestedness. The coffee sites showed lower beta diversity, driven primarily by marked species turnover. Dissimilarity was greater in conventional cacao and coffee plantations compared to agroforestry systems. Cacao sites showed evident clustering, while coffee sites overlapped. Turnover was greater between agroforestry crops *vs.* forest controls in Cacao treatments and conventional *vs.* forest controls in Coffee treatments, this suggests that agroecological practices favor the maintenance of a beetle community more similar to that of the native forest. These differences might be due to anthropogenic disturbances, as dung beetles are very susceptible to environmental variability (Arellano, Leon-Cortes & Halffter, 2008; Costa et al., 2017; Noriega et al., 2021a). We found that the differences in species assemblages are mainly due to turnover in cacao treatments. These differences are not random but are likely due to changes in soil use, which change soil compaction drastically, affecting dung beetles’ nesting and distribution, and determining how species adapt to their habitats (Püttker et al., 2015). However, other variables may be playing an important role that were not quantified, such as forest cover or the food supply of local mammals, which would be important to include in future studies.

Total abundances and biomass were similar for conventional crops and agroforestry coffee, which could be an artifact of recent anthropogenic disturbance (*i.e*., agroforestry) and selective logging, compromising the biological assemblages. Agroforestry systems experience multiple axes of high disturbance, including the removal of a large proportion of plant diversity, which negatively affects insect diversity. Thus, these assemblages can also contain high numbers of species adapted to open areas. Biomass from Cacao treatments was higher in control forests, followed by agroforestry systems, and then lowest on conventional crops (Fig. 5). This is a consequence of the fact that Cacao agroforestry management sites are comprised of larger species, which usually dwell inside forests. Conventional Cacao crops, on the other hand, are characterized by a high abundance of small specimens, like *C. delicatulus*, and these are species well adapted to open areas and usually found in very low numbers inside forests. Our results indicate that agroforestry systems are likely to be useful for preserving big-size species such as *Deltochilum* spp. and *Dichotomius* spp., which use the food efficiently, improving their ecosystem services (Nichols et al., 2007; Nichols et al., 2009; Nichols et al., 2009; Carvajal, Villamarín-Cortez & Ortega, 2011).

Approximately 90% of the terrestrial surface of the earth is outside of reserves and is used or managed by human beings in one way or another (Western & Pearl, 1989), which is also true in Ecuador’s Andean-Chocó region, where extensive mono-crops take place, creating an impact on biodiversity and ecological services provided by insects. Our research supports and has established that agroforestry management practices could ameliorate loss, restore, and maintain insect diversity by exploiting the complementarities and synergisms that result from the combination of crops and forests.

## Conclusions

Agroforestry systems involving cacao and coffee, exhibited dung beetle diversity levels comparable to adjacent reference forests, retaining a significant proportion of forest-associated species, such as *Deltochilum* and *Dichotomius*, which are crucial for ecosystem functions. In contrast, conventional monocultures supported low diversity and were dominated by stress-tolerant species like *Canthon delicatulus*, adapted to open and disturbed habitats.

Species turnover was the primary driver of beta diversity in cacao agroforestry systems, reflecting habitat heterogeneity and niche differentiation. In coffee systems, nestedness played a larger role, suggesting species loss in more disturbed environments. These patterns indicate that agroforestry systems can maintain compositional uniqueness, while conventional systems homogenize assemblages.

Dung beetle diversity was consistently higher during the wet season across all habitat types, emphasizing the importance of seasonal variations in ecological studies. Agroforestry systems mirrored the seasonal patterns observed in forests, further underscoring their role as biodiverse habitats.

Forests adjacent to agroforestry systems had the highest dung beetle biomass, followed by agroforestry plots, with conventional crops showing the lowest values. Larger-bodied species, which contribute disproportionately to ecosystems services, were more prevalent in agroforestry systems, whereas conventional crops favored smaller, generalist species.

Agroforestry systems act as ecological corridors, facilitating species movement between forest fragments and mitigating the negative impact of habitat fragmentation. Their ability to support diverse and functionally important dung beetle assemblages makes them valuable for biodiversity conservation and sustainable land-use planning.

This study demonstrates that agroforestry systems in the Andean Chocó region are not only viable for agriculture, but also essential for conserving dung beetle diversity and the ecosystem services they provide. By integrating agroforestry into regional conservation strategies, Ecuador can safeguard its unique biodiversity while promoting sustainable development.

## Supplemental Information

10.7717/peerj.21163/supp-1 Supplemental Information 1 Pitfall Design Pitfall trap placement in 25 × 25 m sampling plots

10.7717/peerj.21163/supp-2 Supplemental Information 2 R code for beta diversity analysis

10.7717/peerj.21163/supp-3 Supplemental Information 3 Dung beetle abundance and biomass R code

10.7717/peerj.21163/supp-4 Supplemental Information 4 Dung Beetle Seasonality Seasonality and ETA square analysis

10.7717/peerj.21163/supp-5 Supplemental Information 5 Dung beetles data set Data set for all dung beetles collected in all sites and seasons

## Additional Information and Declarations

### Field Study Permissions

The following information was supplied relating to field study approvals (i.e., approving body and any reference numbers): Field collections were permitted by the Ministry of the Environment under the permit No. MAE-DNB-CM-2016-0045.

### Data Availability

The following information was supplied regarding data availability: The code and raw data are available in the Supplemental Files.
