# Supplementary figures and images for: Agroforestry benefits on dung beetle diversity of the Andean-Chocó region in Ecuador
Source: PeerJ. 2026 Apr 23;14:e21163. doi: 10.7717/peerj.21163 (PMC13110650; doi:10.7717/peerj.21163)

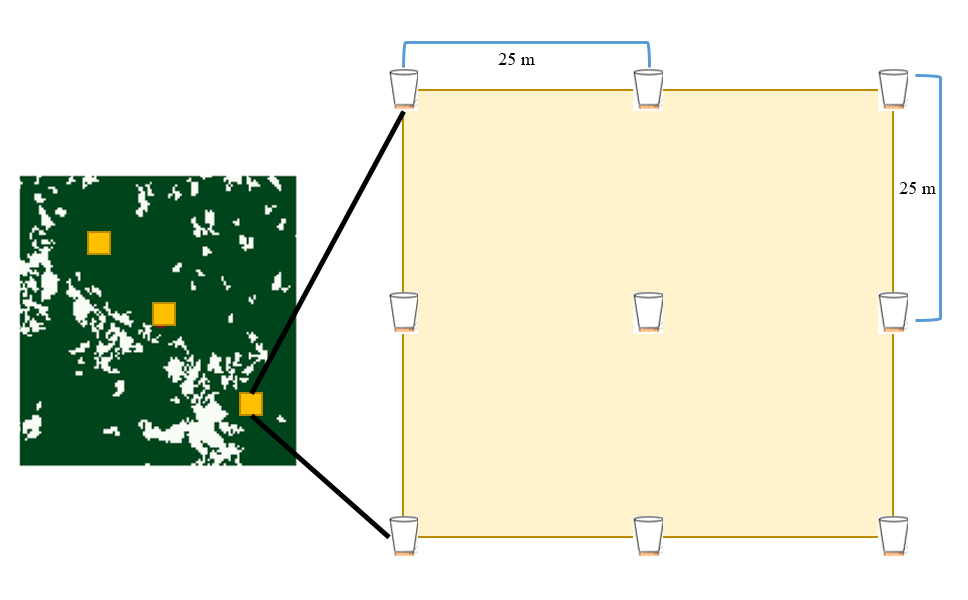

Supplement: Supplemental Information 1 — Pitfall trap placement in 25 × 25 m sampling plots [file peerj-14-21163-s001.png]
